# A case of multiple myeloma with AL amyloidosis showing giant Russel bodies

**DOI:** 10.1002/jha2.678

**Published:** 2023-03-20

**Authors:** Heather Mcphaden, Habib M. Razavi

**Affiliations:** ^1^ Division of Haematology Department of Medicine University of British Columbia Vancouver British Columbia Canada; ^2^ Division of Haematopathology and Transfusion Medicine Fraser Health Authority and University of British Columbia Vancouver British Columbia Canada; ^3^ Department of Pathology and Laboratory Medicine University of British Columbia Vancouver British Columbia Canada

**Keywords:** amyloidosis, multiple myeloma, Russel bodies

1

A 67‐year‐old male patient presented to our hospital with streptococcus pneumonia and a neck phlegmon. As part of an initial workup, a review of an intravenous immunoglobulin order for a possible secondary hypogammaglobinaemia and pneumosepsis showed low IgA and IgM subsets but overall increased IgG class proteins (0.08, 0.3, and 22.8 g/L respectively). The IgG class subset analysis showed decreased IgG class 2–4 but elevated IgG class 1 levels (28.7 g/L). For lack of bona fide hypogammaglobinemia, intravenous immunoglobulin therapy was denied, and further workup including a bone marrow biopsy was undertaken. Patient also presented with secondary erythrocytosis (Haemoglobin 195, janus kinase 2 [Jak2] negative, with increasing erythropoietin [EPO] level), attributed to severe sleep apnea and asthma. A serum electrophoresis (SPEP) showed an IgG lambda monoclonal protein (19 g/L) with elevated serum lambda free light chain (588.5 mg/L) with a marked concomitnat background gamma globulin suppression. A bone marrow biopsy showed scanty background residual trilinear hematopoiesis with an increase in lambda clonal plasma cells (90% of cellularity). Specifically the bone marrow aspirate showed frequent inclusions overlaying the nucleus (Dutcher bodies) and cytoplasm (Russel bodies) of the plasma cells (Figure 1, panel A, white and black arrows respectively, stained by May‐Grünwald‐ Giemsa x 40 objective). Many of the inclusions seen were smaller leaving the cellular morphology intact. Having said this, within the aspirate particles, large/giant inclusions were seen (panel B, aspirate granule x40 magnification). Moreover, a trephine biopsy showed many large spherical bodies which showed cytoplasmic ballooning and resulted in complete cellular effacement and displacement of the nucleus (Figure 1, panels C–E Hematoxylin and Eosin stain x40 (C) and x50 (D,E) magnification respectively; black arrows point to overstuffed plasma cells with eccentric nuclei). CD138/lambda stains confirmed that these were not cell‐free but were inclusions within the plasma cell cytoplasm (Figure 1, panels F and G, CD138 and lambda stains respectively, x20 magnification). Lastly a Congo red stain for amyloid showed salmon pink deposits with apple green birefringence under polarized light (panels H&I showing a bone marrow blood vessel, Congo red ×20 magnification). With a confirmed diagnosis of plasma cell myeloma the patient was treated with cyclophosphamide, bortezomib and dexamethasone therapy, forgoing any intravenous immunoglobulin. The first description of intracytoplasmic inclusion in multiple myeloma were provided by Russel (1890) and subsequently termed Russel bodies. Mott cells refer to plasma cells with multiple inclusions such as Russel bodies. Dutcher bodies refer to cytoplasmic inclusions that overlay the nucleus. These cytoplasmic inclusions have been observed in both reactive and malignant plasma cells. They represent accumulations of mucopolysaccarides and immunoglobulin, which highlights the common nature of these inclusions. There is no established prognostic significance of such inclusions. One study by Jiang et al. reveals a correlation between the presence of Dutcher bodies and t(4;14), which is an adverse prognostic marker in multiple myeloma.

**FIGURE 1:**
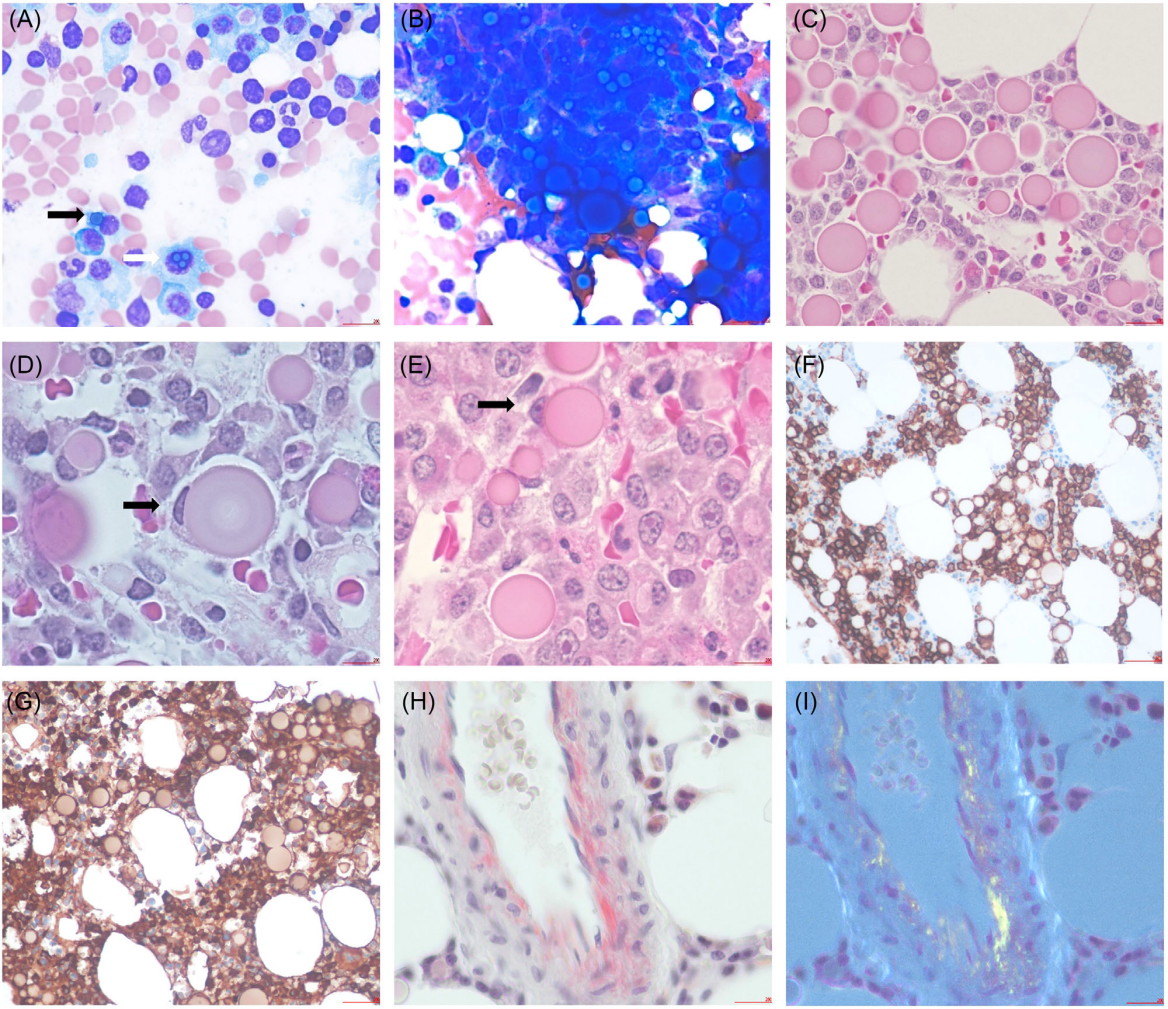
Bone marrow aspirate and biopsy show >90% lambda clonal plasmacytosis in keeping with the diagnosis of plasma cell myeloma. Numerous giant Russel bodies are present in both the aspirate and biopsy (panels A–E). CD138 and lambda stains (panels F and G) show that these inclusions are not cell free and represent overstuffed malignant plasma cells. Panels H and I show concomitant presence of congophilic deposits in keeping with AL amyloidosis. Abbreviation: AL, amyloid light chain.

## CONFLICT OF INTEREST STATEMENT

The authors declare that there is no conflict of interest that could be perceived as prejudicing the impartiality of the research reported

## FUNDING INFORMATION

The authors received no specific funding for this work.

## ETHICS STATEMENT

There are no patient identifiers in this study. Verbal consent has been obtained at the time of bone marrow biopsy.

## Data Availability

All data have been presented and are available for review.

